# Agronomic performance of *Populus deltoides* trees engineered for biofuel production

**DOI:** 10.1186/s13068-017-0934-6

**Published:** 2017-11-30

**Authors:** David Macaya-Sanz, Jin‐Gui Chen, Udaya C. Kalluri, Wellington Muchero, Timothy J. Tschaplinski, Lee E. Gunter, Sandra J. Simon, Ajaya K. Biswal, Anthony C. Bryan, Raja Payyavula, Meng Xie, Yongil Yang, Jin Zhang, Debra Mohnen, Gerald A. Tuskan, Stephen P. DiFazio

**Affiliations:** 1 Department of Biology, West Virginia University, Morgantown, WV 26506 USA; 2 BioEnergy Science Center and Biosciences Division, Oak Ridge National Laboratory, Oak Ridge, TN 37831 USA; 3 Department of Biochemistry and Molecular Biology, University of Georgia, Athens, GA 30602 USA; 4 Complex Carbohydrate Research Center, University of Georgia, Athens, GA 30602 USA

**Keywords:** *Populus*, Transgenic, Field trial, Insects, Frost, Yield, Phenology, Cell wall, Biofuel

## Abstract

**Background:**

One of the major barriers to the development of lignocellulosic feedstocks is the recalcitrance of plant cell walls to deconstruction and saccharification. Recalcitrance can be reduced by targeting genes involved in cell wall biosynthesis, but this can have unintended consequences that compromise the agronomic performance of the trees under field conditions. Here we report the results of a field trial of fourteen distinct transgenic *Populus deltoides* lines that had previously demonstrated reduced recalcitrance without yield penalties under greenhouse conditions.

**Results:**

Survival and productivity of the trial were excellent in the first year, and there was little evidence for reduced performance of the transgenic lines with modified target gene expression. Surprisingly, the most striking phenotypic effects in this trial were for two empty-vector control lines that had modified bud set and bud flush. This is most likely due to somaclonal variation or insertional mutagenesis. Traits related to yield, crown architecture, herbivory, pathogen response, and frost damage showed few significant differences between target gene transgenics and empty vector controls. However, there were a few interesting exceptions. Lines overexpressing the *DUF231* gene, a putative *O*-acetyltransferase, showed early bud flush and marginally increased height growth. Lines overexpressing the *DUF266* gene, a putative glycosyltransferase, had significantly decreased stem internode length and slightly higher volume index. Finally, lines overexpressing the *PFD2* gene, a putative member of the prefoldin complex, had a slightly reduced volume index.

**Conclusions:**

This field trial demonstrates that these cell wall modifications, which decreased cell wall recalcitrance under laboratory conditions, did not seriously compromise first-year performance in the field, despite substantial challenges, including an outbreak of a stem boring insect (*Gypsonoma haimbachiana*), attack by a leaf rust pathogen (*Melampsora* spp.), and a late frost event. This bodes well for the potential utility of these lines as advanced biofuels feedstocks.

**Electronic supplementary material:**

The online version of this article (doi:10.1186/s13068-017-0934-6) contains supplementary material, which is available to authorized users.

## Background

The considerable energy contained in plant cell walls is an attractive target for the biofuels industry. Cell walls contain approximately 70% of the carbon fixed by plants globally, and constitute a relatively untapped global energy resource [1]. One of the main barriers for the utilization of lignocellulosic biomass for biofuel production is the recalcitrance of plant cell walls to chemical and enzymatic deconstruction, which is a necessary step to release sugars for subsequent conversion to fuels. Recalcitrance is primarily a consequence of the plant packaging carbohydrates in forms that are inaccessible to degradation by chemical and biological agents. Recalcitrance can be a feature of the cellulose polymer itself, which is packaged in tightly interconnected fibers that can be organized into crystalline sheets that themselves are relatively inaccessible to cellulolytic enzymes [1, 2]. These fibers occur within a largely hydrophobic matrix of lignin, which also contributes to recalcitrance. Cellulose, a polymer of 6-carbon glucose molecules (C6) is also entwined with and bound to hemicelluloses, principally xylans in angiosperms, which are mainly comprised of 5-carbon sugars (C5) that are not as readily converted to fuel as the 6-carbon sugars like the glucose monomers that make up the cellulose chains [1–3]. The hemicelluloses and other non-cellulosic cell wall polymers may also contribute to recalcitrance. This structural complexity of the wall makes bioconversion of lignocellulosic biomass to liquid fuels challenging and expensive.

Release of sugars for subsequent fermentation to fuels can be achieved by a series of separate steps aimed at (1) physically reducing the size of the biomass to maximize surface-to-volume and/or weight-to-volume (density) ratio; (2) pretreatment with heat and chemicals such as dilute acids to enhance porosity; (3) treatment with biocatalysts to break down the cross-linkages between cellulose microfibrils and the cell wall matrix; and (4) subsequent hydrolysis with industrial enzymes such as cellulases to produce the sugars [4, 5]. These processes are expensive due to the large energy requirements and the cost of the enzymes. An attractive alternative is consolidated bioprocessing (CBP), which ideally involves minimal pretreatment, and integrates the production of the hydrolytic enzymes with the fermentation step [6]. Major technological advances are however needed to enable CBP. Ideally the process would involve microbes that can hydrolyze cellulose and hemicellulose from minimally processed biomass feedstock and utilize both C5 and C6 sugars in fermentation under harsh conditions and with minimal inhibition from the fermentation products [7, 8]. Major advances have been achieved in recent years, such as with recent breakthroughs in optimizing organisms such as *Clostridium thermocellum* [9] and *Caldicellulosiruptor bescii* [10] for CBP utilization.

Another potential component of efficient biofuel production is the development of biomass feedstocks with cell walls that can be readily deconstructed to yield fermentable sugars [4, 11, 12]. One way to achieve this is to manipulate the expression of genes involved in the biosynthesis of cell walls using genetic transformation. Major phenotypic targets to reduce recalcitrance include: (1) altering cellulose biosynthesis to increase cellulose content and reduce crystallinity; (2) altering hemicellulose composition to decrease H bonding with cellulose; (3) altering enzymes in the phenylpropanoid pathway to reduce lignin content or composition to reduce covalent cross-linkages; and (4) altering the structural proteins in the cell wall or and/or cortical microtubules [1, 3, 5]. To this end, the Department of Energy’s Bioenergy Science Center (BESC) has targeted over 500 distinct genes for overexpression and/or knockdown using *Agrobacterium*-mediated transformation of *Populus deltoides*. These transformants have been intensively screened using high-throughput assays to evaluate cell wall composition [13] and sugar release from wood with minimal pretreatment [14]. This evaluation has resulted in the identification of 14 genes that, when overexpressed or knocked down, result in biomass with reduced recalcitrance and no yield penalty based on greenhouse and growth chamber trials (Table 1). The selected genes fall into seven categories, based on the pathways or characteristics that they are expected to affect: (1) phenylpropanoid biosynthesis (*CAD*, *EPSPS*); (2) cellulose biosynthesis (*IQD10*); (3) noncellulosic cell wall polysaccharide biosynthesis (*GAUT12*); (4) cell wall glycoproteins (*EXT1*,*EXT2*); (5) cell wall modifiers (*DUF231*, *DUF266*, *P4HA1*, *RWA2*,*SHMT*); (6) cortical microtubule formation (*PFD2*); and (7) transcription factors controlling enzymes involved in cell wall biosynthesis (*HB3*,*VND6*).

**Table 1:** Description of genes targeted in this study

Name	Gene^a^	Description	Type^b^	Sugar Release^b^	Yield^b^	Ramets^c^
*CAD*	*Potri.009G095800*	Cinnamyl alcohol dehydrogenase, catalyzes the formation of coniferyl or coumaryl alcohol from their respective aldehydes [58]	KD	+	+	12(C), 12(T)
*DUF231*	*Potri.009G072800*	Domain of unknown function, a member of the Trichome Birefringence-Like (TBL) gene family, possibly responsible for *O*-acetylation of hemicelluloses [49]	OE	+	+	12(C), 12(T)
*DUF266*	*Potri.011G009500*	Domain of unknown function, possibly acting as a glycosyltransferase [53]	OE	+	+	12(C), 5(C), 5(T)
*EPSPS*	*Potri.002G146400*	5-enolpyruvylshikimate-3-phosphate synthase, key enzyme in biosynthesis of aromatic amino acids [59]	OE	+	=	12(C), 12(T)
*EXT1*;*EXT2*	*Potri.001G020100* *Potri.005G190100*	Extensin, a basic hydroxyproline-rich glycoprotein localized to the cell wall [60]	KD	+	+	12(C), 12(T); EXT2: 12(T)
*GAUT12*	*Potri.001G416800*	Galacturonosyltransferase targeted to Golgi and involved in xylan and homogalacturonan biosynthesis [61]	KD	+	+	12(C), 12(C), 12(T)
*HB3*	*Potri.011G098300*	Transcription factor belonging to the HDZIPIII family, with high expression in xylem tissue [62]	KD	+	+	12(C), 14(T)
*IQD10*	*Potri.001G375700*	Calmodulin-binding protein with IQ amino acid-rich region with high expression in tension wood [63]	KD	+	+	11(C), 13(T)
*P4HA1*	*Potri.017G075300*	Prolyl 4-hydroxylase alpha subunit, hydroxylation of proline residues, potentially in hydroxyproline-rich glycoproteins in the cell wall [64]	OE	+	=	12(C), 12(T)
*PFD2*	*Potri.008G153900*	Probable Prefoldin 2 protein. A heterohexameric chaperon protein that binds to actin, tubulin, and other proteins, possibly affecting the cortical spindle [55]	OE	+	+	12(C), 13(T)
*RWA2*	*Potri.010G148500*	Reduced wall acetylation 2, catalyzes *O*-acetylation of cell wall polysaccharides [50]	OE	+	+	13(C), 12(T)
*SHMT*	*Potri.001G320400*	Serine hydroxymethyltransferase, reversible conversion of Ser and tetrahydrofolate to Gly and 5,10-methylene tetrahydrofolate, providing a major 1-carbon source for the cell [65]	OE	+	+	12(C), 12(T)
*VND6*	*Potri.015G127400*	Vascular-related NAC-domain Protein 6, transcription factors involved in xylem vessel differentiation [66]	OE	-	-	12(C), 13(T)

*KD* knockdown of gene expression using RNAi, *OE* overexpression of target gene using a constitutive promoter (*UBIQUITIN3*)

^a^Gene model name based on phytozome [67] version 3.0 of the *Populus trichocarpa* genome

^b^Sugar release [14] and yield refer to performance relative to controls in greenhouse trials prior to the field trial

^c^The number of replicates (ramets) included in the statistical analyses for TOP (T) and comparator (C) lines. For *DUF266* and *GAUT12* there were two comparator lines, which are listed separately

While demonstration of enhanced performance under greenhouse conditions is a significant achievement, it is essential to evaluate the performance of these lines in replicated field trials under realistic field conditions, where results are often qualitatively different [15]. This is particularly important in the case of traits that affect cell wall structure and composition, as the cell wall plays a crucial role in resisting the pervasive biotic and abiotic stresses that predominate under field conditions [11, 16, 17]. Furthermore, although there is ample evidence that transgene expression can be stable over many years and through multiple rounds of vegetative propagation [18–20], there are also many examples of differential performance of transgenic trees under field and laboratory conditions [16].

One illustrative example is the case of the 4-hydroxycinnamoyl-CoA Ligase (*4CL*) gene in *Populus*. This enzyme catalyzes a key step in the lignin biosynthetic pathway, responsible for the conversion of *p*-coumaric acid to *p*-coumaroyl CoA [21]. Knocking down expression of this gene in *Populus tremuloides* led to reduced lignin and enhanced growth under greenhouse conditions [22]. Although the lignin reduction has mostly been consistent in subsequent field trials in this and other genetic backgrounds, growth has typically been reduced relative to wild-type under most field conditions [23, 24]. This impaired performance was apparently due to problems with vessel collapse under water stress and partial occlusion of vessels by tyloses and phenylpropanoid deposition in the transgenics [23, 25]. Clearly evaluation of transgenics with altered cell wall properties under field conditions is essential, and should include evaluation of growth as well as responses to biotic and abiotic stressors [16].

Here we describe the results of a field trial of 36 transgenic lines of *Populus deltoides* representing modification of 14 genes that previously satisfied an intensive screening process under greenhouse and growth chamber conditions. We show that, by and large, the transgenic lines perform equally well as controls in terms of biomass productivity, crown form, and biotic and abiotic stress tolerance during the first year. This is an important milestone in the development of these improved biofuel feedstocks.

## Methods

### Generation of transgenic lines

Gene targets (Table 1) were initially identified using a combination of data mining approaches [26], expression studies of tissues undergoing enhanced cellulose synthesis [27–29], analysis of activation-tagged lines with altered cell wall characteristics [30], and association genetics analyses of wild populations of *P. trichocarpa* [31]. *Agrobacterium tumefaciens*-mediated transformation was performed in *Populus deltoides* clone WV94 from Issaquena County, MS by Arborgen, LLC as described previously [32]. For overexpression (OE) constructs, full-length transcripts were amplified from either *P. deltoides* or *P. trichocarpa* and inserted 3′ of a constitutive promoter (*UBQ3* from *Arabidopsis thaliana*) and 5′ of the NOS terminator from *Agrobacterium tumefaciens*. In the case of knockdown (KD) constructs, a unique fragment of the coding sequence of the target gene was cloned as an inverted repeat separated by an intron cloned from the *CHALCONE SYNTHASE* gene of *Petunia hybrida*, with the same promoter and terminator as described above. Empty vector controls (seven independent lines) were produced simultaneously using identical methods and vectors, minus the transgenes. These plants were propagated from tissue culture and subsequently from greenwood cuttings, together with non-transformed ramets of clone WV94 that had not been through tissue culture (wild type controls). The plants were propagated in a greenhouse at Oak Ridge National Laboratory (Oak Ridge, TN) at 25 °C and 16 h day length. All lines were evaluated in the greenhouse for growth and form, and analyzed for lignin content, syringyl:guaiacyl (S:G) ratio, and sugar release using methods described previously [13, 33]. The top-performing line (referred to below as the TOP line) and at least one transgenic comparator line were selected for each target gene, except for *EXT2*, for which only the TOP line was available.

### Field trial establishment and design

The field trial was established near Morgantown, WV under USDA APHIS permit 15-047-101. The site has mildly sloped topography and had mostly been under hay cultivation for at least a decade prior to the trial. Site preparation was conducted during the spring and summer of 2015 and included treating with herbicide (Glyphosate and Clopyralid (Stinger^®^, Dow AgroSciences)), grading, plowing, and tilling. The site was then left fallow for a year, with repeated herbicide sprays to exhaust the seed bank. The site was then tilled again in the spring of 2016 prior to transplanting the rooted cuttings for all *P. deltoides* lines, comparators, and controls.

Rooted cuttings were planted on June 20, 2016, consisting of 512 ramets in the WV94 background. All lines had at least 11 clonal replicates, with the exception of two of the DUF266 lines, which only had 5 replicates. At the time of establishment the plants averaged 76.4 ± 10 cm (SD; range 45–99 cm) tall and had been maintained at tight spacing in Leach Tubes (3.8 cm in diameter, 14.0 cm deep). The trees were planted at a spacing of 1.2 m within rows and 3 m between columns, with columns in an approximately North–South orientation. There were 16 trees per column and 32 columns. Trees were randomized within blocks, which corresponded to approximately 2.5 columns each. The plantation was surrounded by a single border row consisting of extra transgenic and nontransgenic trees from the same background. Each tree was planted in the center of a 91 × 91 cm porous mat to control weed competition (VisPore^®^ Tree Mats, Forestry Suppliers, MS, USA), staked and encircled by a 45-cm plastic tree collar to protect from rodents (Protex^®^ Tree Collars, Forestry Suppliers, MS, USA). All trees were supported by a 1 m bamboo stake to prevent lodging due to high wind. The entire trial was surrounded by an electric fence to exclude large mammals.

All trees received irrigation using a T-tape drip irrigation system with 20-cm spacing between emitters (Aqua-Traxx). Trees were irrigated for 2 h per night for the first 2 months after establishment. This was reduced to 1 h on August 30 and to 30 min on September 5. Plants were fertilized twice with approximately 5 g of 19:19:19 N:P:K fertilizer (ca. 50 kg/ha) on July 30 and again on August 15. Granules were poured directly into the tree collars. Weeds were controlled by periodic sprays of Glyphosate and Clopyralid around the porous mats and by manual removal within the tree tubes, as needed.

### Phenotyping and trait measurements

In order to evaluate the field performance of the 37 transgenic lines, 17 phenotypes were measured. These traits were selected to account for (1) yield and growth, (2) crown architecture, (3) vegetative phenology, and (4) response to an array of biotic and abiotic stressors (Table 2).

**Table 2:** Phenotypes measured in the field trial

Phenotypes	Units	Mean	SD^a^	*r* ^2 b^	*P*-value^c^
Growth and yield					
Total height	cm	207	16	0.558	*2.23E−12*
Internode length	cm	14.8	1.8	0.373	6.92E−03
Height growth	cm	130	17	0.317	*3.28E−06*
Quadratic mean diameter	mm	23.2	2.9	0.602	*5.86E−05*
Volume index	m^3^	0.362	0.108	0.670	*1.87E−05*
Crown architecture					
Height to first branch	cm	117	15	0.297	*5.03E−06*
Number of branches	Counts	13.3	4.0	0.286	*6.85E−09*
Stem sinuosity	Score: 0–4	1.21	0.86	0.028	3.89E−02
Stem length-height ratio	NA	0.988	0.019	0.187	4.70E−02
Apical index	NA	1.40	0.13	0.257	1.65E−02
Trunk section eccentricity	NA	0.215	0.097	0.069	6.00E−01
Vegetative phenology					
Bud set	Score: 1–6	3.04	0.17	0.007	*1.39E−74*
Bud flush	Score: 1–6	5.18	0.35	0.189	*2.55E−09*
Stress response					
Frost damage	Score: 0–3	1.93	0.26	0.385	1.50E−01
*Melampsora* severity	Score: 0–4	2.99	0.12	0.064	3.51E−01
Overall herbivory	Score: 1–10	2.41	1.69	0.100	8.56E−01
Twig borer incidence	Counts	2.79	1.86	0.096	6.18E−01

*NA* not applicable for dimensionless trait

^a^The standard deviation (SD) of observed values

^b^Coefficient of determination, *r* ^2^, between the observed values and the values predicted by the TPS models, an indicator of the degree of spatial-dependent variation in the trait

^c^Significance level, *P* value, of the one-way ANOVAs for all lines (*k* = 37; including the seven empty-vector control lines and the wild type)

All measurements were performed on November 12–13, 2016 after all trees had become dormant, except as noted. Yield was estimated by (1) total height: the perpendicular distance between the ground and the apical bud; (2) relative height growth: the difference between the total height and the height of the plants at establishment; (3) quadratic mean diameter: the quadratic mean of the largest trunk transverse section axis and its perpendicular axis; (4) the volume index: the volume of a virtual cylinder with dimensions of total height and quadratic mean diameter; and (5) internode length: the total length of four internodes on the dominant stem leader. The four internodes were selected from the middle portion of the current year growth, where the size of the internodes was more uniform than at the beginning and end of the growing season.

To depict tree crown architecture, we measured (1) height to the first branch: the perpendicular distance between ground and the lowest branch on the tree; (2) number of branches: the number of primary branches on the stem; (3) stem sinuosity: a perceptual score from 0 (straight trunk) to 4 (heavily sinuous trunk); (4) stem length-height ratio: the ratio between the actual trunk length and the total height (defined as above); (5) the apical index: the ratio between the diameter of the apical stem, and of the mean of six lateral branch twig diameters, measured at the base of the 2017 new growth; and (6) trunk section eccentricity: the mathematical first eccentricity of the virtual ellipse created by the largest trunk transverse section axis and its perpendicular axis, as measured above.

Vegetative phenology was appraised by means of (1) bud set stage of the apical bud on October 11, 2016 using a visual scale ranging from 1 (actively growing) to 6 (bud completely set) [34]; and (2) bud flush stage on April 12, 2017, scoring from 1 (bud still dormant) to 6 (actively growing with fully developed leaves).

Finally, response to biotic and abiotic stress was evaluated by quantifying the incidence of three pervasive stressors in the field trial and a general estimation of arthropod grazing pressure. Frost damage was estimated on May 18, 2017 after an episode of late frost, using a visual scale of damage in the apical shoot from 0 (no necrosis) to 3 (apical meristem macroscopically detrimentally affected). *Melampsora* spp. severity was also measured with a visual score from 0 (no macroscopic symptoms) to 4 (> 50% canopy defoliation). Overall insect herbivory was scored from 0 to 10 based on the proportion of leaf area affected by feeding. Finally, incidence of the cottonwood twig borer *Gypsonoma haimbachiana* was assessed by counting the total number of larval holes made in six lateral branches plus the apical stem.

### Statistical analyses

Although the experiment was designed to minimize environmental sources of variance, most of the traits studied are very influenced by microsite heterogeneity. To account for this, we modeled spatial variation of each trait using a thin plate spline (TPS) algorithm, using the R package module ‘fields’ [35]. The residuals of the models were retrieved and rescaled to the overall trait means to generate trait estimates with minimized spatial variation.

We performed an overall one-way ANOVA for each trait (*k* = 37), using transgenic line as factor. This analysis included the nine empty-vector control lines as well as the untransformed wild type WV94. To test for non-target effects of transformation, we performed one-way ANOVA for each trait using just the wild type and the empty-vector control lines as factors (*k* = 8). Finally, to test the actual effects of the transgenes in the WV94 background, we performed specific contrasts between the empty vector control lines and the lines containing the target gene constructs, as follows. First, to avoid an unbalanced contrast, we randomly selected a subset of 15 individuals of the empty-vector lines to be used as controls. We excluded lines EV1 and EV9 because these lines had clear evidence of somaclonal variation (see Results). Second, we tested for trait mean significant differences (one-way ANOVA) for all lines per construct together with the empty-vector control subset (*k* = 2–4, depending on the construct). Finally, whenever the ANOVA was significant, we conducted a Tukey’s HSD test to identify the pairs of lines that were significantly different. To account for false positive rate due to multi-testing, we restricted the significance threshold using Bonferroni correction.

## Results and discussion

### Trial establishment

**Fig. 1:**
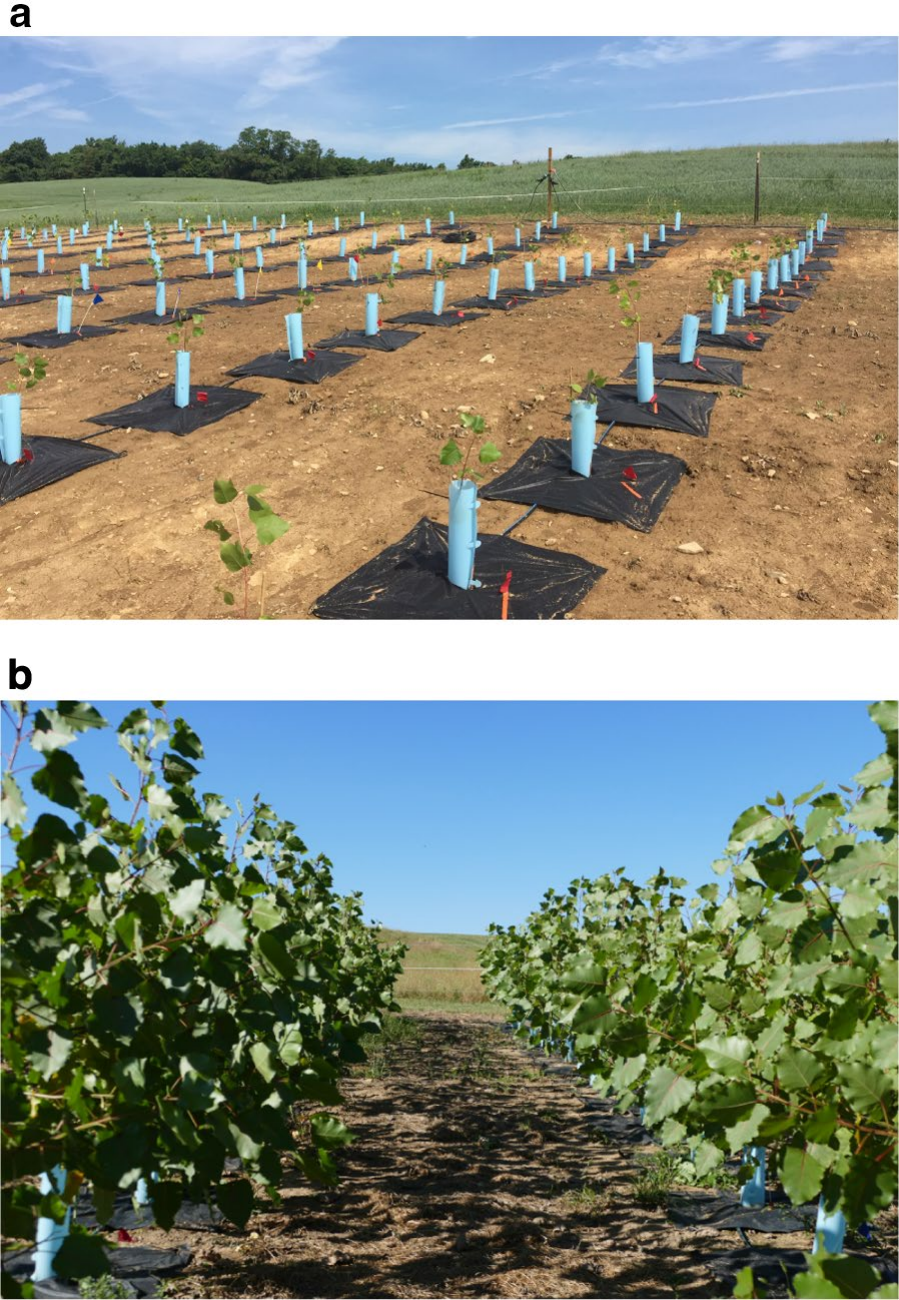
Pictures of the trial **a** immediately after establishment on June 20, 2016, and **b** on October 3, 2016

The establishment of the trial was excellent (Fig. 1a). The survival rate was 100% and the trees had negligible transplant shock prior to resumption of growth. The overall productivity was high, with an average uncorrected height of 207 ± 16 cm (SD) and volume indexes of 0.362 ± 0.108 m^3^ (SD) at the end of the growing season (Fig. 1b). This growth was comparable to that reported in a transgenic trial in Belgium in the *Populus tremula* × *alba* cv. “717–1B4” background, but our trial had substantially lower variance [36]. The Belgian trial was also for a single growing season, and heights were approximately 225 ± 25 cm (SD). Stem dry weight, which should be proportional to volume index, was approximately 60 ± 20 g (SD) within genotypes. An Anglo-French study revealed similar net growth and slightly higher estimates of SD [37] than herein. Therefore, given the high productivity of poplar trials, 1 year analyses are highly informative of relative performance of transgenic lines [36].

### Thin plate spline correction

**Fig. 2:**
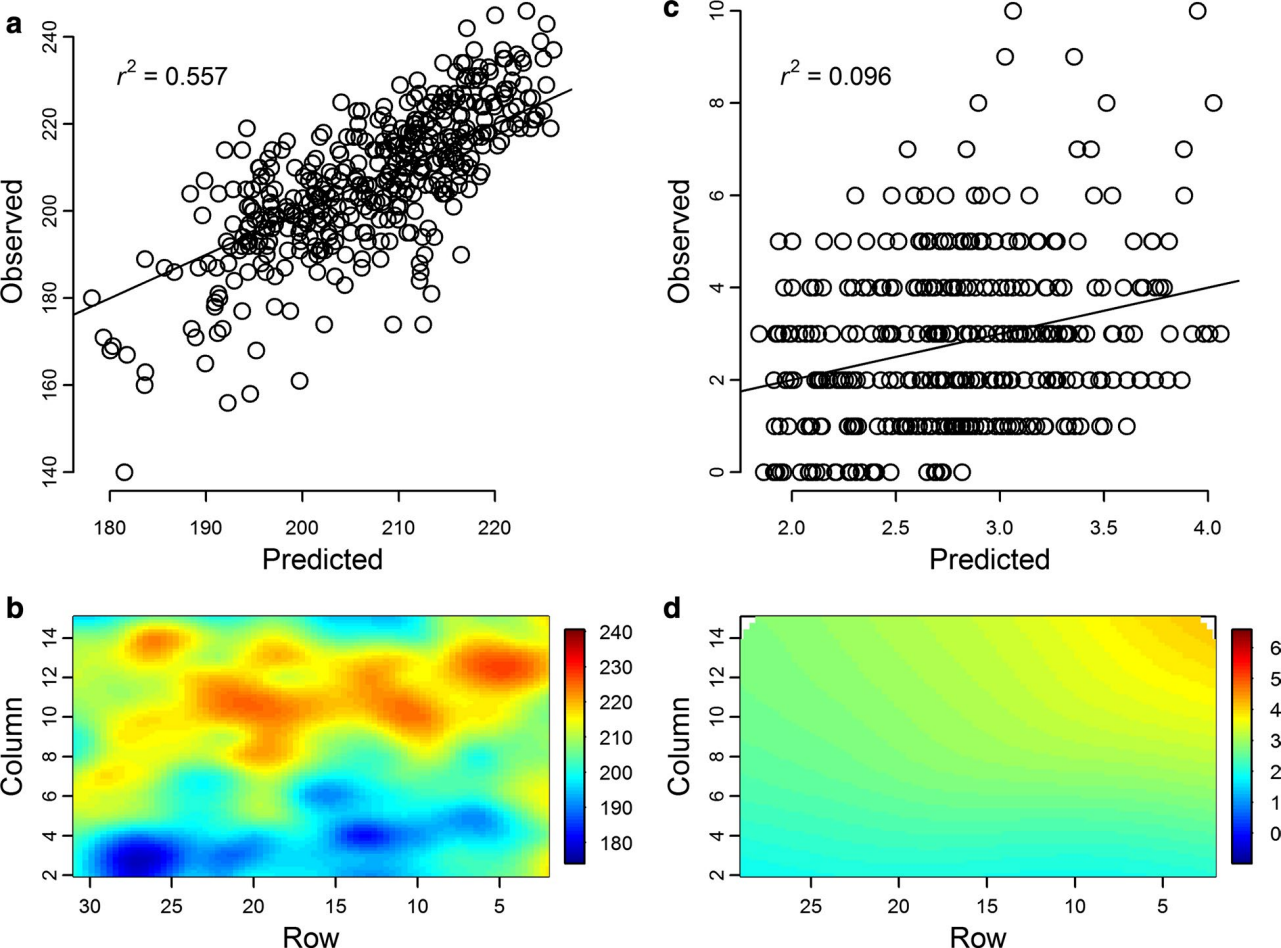
Thin-plate spline correction models. **a** Scatter plot of the total height values predicted by the model versus the observed values with the coefficient of determination (*r* ^2^). Also shown is the 1:1 line. **b** Heatmap of the trial layout with the total height predicted values by coordinate. Note that the color scale ranges from twice the standard deviation over the mean of the observed values to twice the standard deviation below the mean, to reflect the proportion of trait variance accounted for by the model. **c**, **d** Same plots for twig borer incidence

TPS models reduced spatial variation in nearly all measured traits, though to different extents. Traits related to yield and growth had relatively high (predicted-vs-observed *r* ^2^ ranging 0.50–0.70; Fig. 2a, b) or moderate (*r* ^2^ = 0.15–0.40) spatial variability (Table 2). Crown architecture traits were mostly moderately affected by position, except trunk sinuosity and eccentricity, which were lightly affected (*r* ^2^ ≤ 0.10). Regarding vegetative phenology, bud flush was moderately affected by position whereas bud set was the trait with the lowest *r* ^2^ (almost negligible), as expected, given that it is primarily driven by day length rather than temperature [34, 38]. Finally, none of the biotic stressors showed strong position-dependence (*r* ^2^ ≤ 0.10; Fig. 2c, d; Additional file 1), but abiotic stress in the form of frost damage was moderately influenced by position in the field (*r* ^2^ = 0.385; Table 2).

### Trait variance across lines

The overall significance of the trait differences among lines was tested using a one-way ANOVA with *k* = 37 groups (i.e. lines) (Table 2). Interestingly, none of the traits reflecting direct responses to environmental stressors showed significant differences across the line means (ANOVA *P* > 0.15; Table 2). This is despite the fact that there was a serious outbreak of *Melampsora* leaf rust that affected 100% of the trees, attack by the cottonwood stem borer (*Gypsonoma haimbachiana*) that affected 94.2% of the trees, and a late frost event in May 2017 that caused visible damage on 99.9% of the trees.

Conversely, vegetative phenology showed strong differences among lines for both bud flush and bud set (ANOVA *P* < 1E−08). Within crown architecture traits, tests on height to first (highest) branch and number of branches were strongly significant (ANOVA *P* < 1E−05), whereas all other crown architecture traits were marginally or not significant (ANOVA *P* > 0.01). In general, yield trait tests were very significant (ANOVA *P* < 1E−05), with the sole exception of internode length, which was marginally significant (ANOVA *P* = 0.007) (Table 2).

It is worth noting that most of the traits with reduced spatial variation (estimated by the TPS predicted-vs-observed *r* ^2^) also displayed non-significant one way ANOVAs (Table 2). This indicates either that the inter-individual variance was very high (i.e. they are traits with high phenotypic plasticity in the background WV94) or it was very low (i.e. all the individuals have almost the same value). Only bud set did not follow the pattern, with very low spatial dependence but enormous inter-line variance.

### Empty-vector controls

**Fig. 3:**
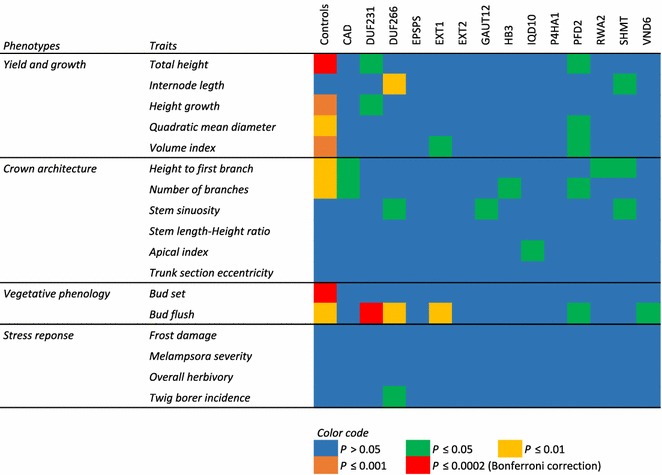
Heatmap of the level of significance of one-way ANOVAs per gene and per trait. Number of groups (*k)* varies as a function of the number of lines per gene: Control tests include the wild type and the seven empty-vector controls (*k* = 8) whereas the target gene tests include the random subsample of empty-vector trees plus the transgenic lines of each gene (from one to three; thus, *k* = 2–4)

**Fig. 4:**
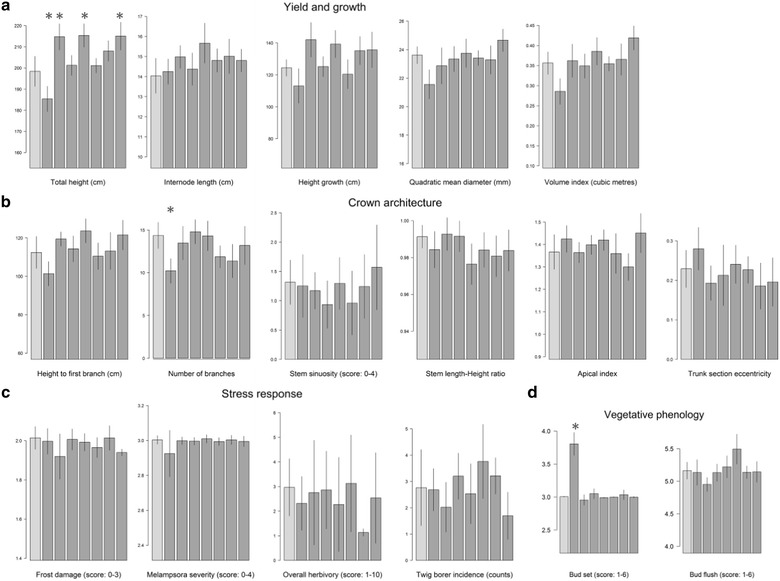
Bar plots of the eight control lines for the measured traits after TPS correction. Traits are indicative of **a** growth and yield, **b** crown architecture, **c** responses to stressors, and **d** vegetative phenology. Wild type WV94 is represented as the light grey bar and the empty-vector controls as the dark grey bars, ordered from EV1 to EV7. Error bars represent 95% confidence intervals. Asterisks indicate Tukey’s HSD mean difference significance (*α* = 0.05) between the marked empty-vector line and the wild type

Empty vector control lines showed highly significant differences among lines for bud set, total height, height growth, and volume index and moderately significant differences for height to the first branch, number of branches, trunk diameter, and bud flush (Fig. 3). *Post hoc* pairwise contrasts between individual lines and the wild type control (Tukey’s HSD) revealed a lack of pairwise significant differences for most traits (Fig. 4a–d). However, lines EV2, EV4 and EV7 had significantly greater height than the wild type, though the differences were not dramatic, amounting to an approximately 6% increase in average height (Fig. 4a). More strikingly, bud set for line EV1 was markedly earlier than for the wild type control line and all of the other empty-vector lines (Fig. 4d). The resulting reduction in growing season ostensibly affected other traits like total height and number of branches as well (Fig. 4a, b). In contrast, line EV5 flushed significantly earlier than four other empty-vector lines, but not than the wild type (Fig. 4d).

Comparing the one-way ANOVAs between the control lines (WT and EVs) and the target gene lines (EV subsample plus the Comparator and TOP lines), significance was in general much larger within the control lines (Fig. 3), reflecting greater inter-line variance for the empty-vector controls compared to the gene vectors. It is well known that tissue culture and organogenesis can generate genetic instability due to cytosine methylation, repeat-induced point mutations, gross chromosomal rearrangements, and retrotransposon activation [39–43]. This somaclonal variation is apparently driven by oxidative stress cascades triggered by tissue culture conditions [44]. Furthermore, the T-DNA insertions of empty vectors could disrupt coding sequences or regulatory elements, thereby causing genetic changes and sometimes observable phenotypic modifications [45]. This process, called insertional mutagenesis, has been well characterized and widely used in functional genomics of model organisms, including plants [46, 47]. Furthermore, the promoters within the empty vectors could activate nearby genes, a fact that has been exploited previously in activation tagging efforts in *Populus* [30, 48]. Which of these different possible phenomena underlie our case remains to be explored further. However, this finding highlights the importance of including several independent empty-vector controls in transgenic filed trials to adequately estimate the background phenotypic variance generated solely by tissue culture and vector insertion and, therefore, appropriately calculate the significance of transgenic gains.

### Transgenic TOP lines

**Fig. 5:**
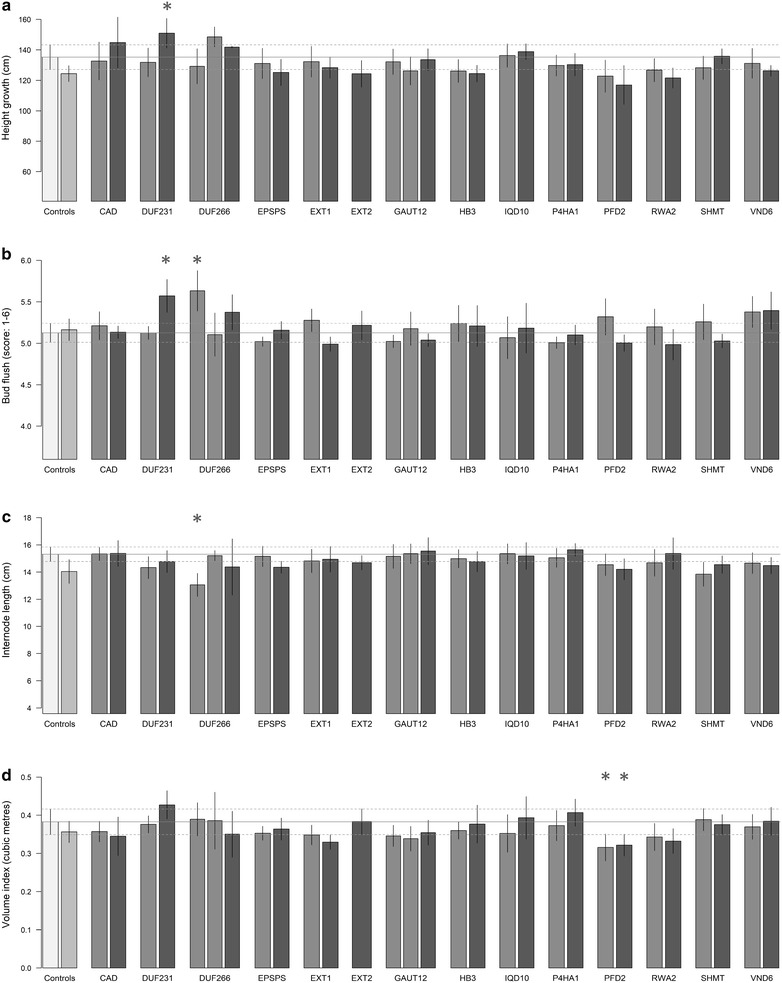
Bar plots of several measured traits after TPS correction for the random subsample of empty-vector trees (very light grey), the wild type (light grey), and the 29 trans-lines grouped by genes. Dark grey indicates Comparator lines and very dark grey indicates TOP lines. Error bars represent 95% confidence intervals. Asterisks indicate Tukey’s HSD mean difference significance (*α* = 0.05) between the marked transgenic line and the empty-vector control. Traits depicted are **a** height growth, **b** bud flush, **c** internode length, and **d** volume index

The effects of the target genes on the measured traits were weak in general, estimated through one-way ANOVAs per gene and trait (number of tests = 238), with lines as groups including the empty-vector random subset as a negative control (*k* from 2 to 4). The main general trend observed was the lack of effect on the four traits related with stress responses (Additional file 2). Only the lines targeting the *DUF266* gene seemed to be slightly affected, with a marginal ANOVA *P* value that could be an artifact of multiple testing (Fig. 3). Trunk section eccentricity was also not affected by any of the transgenes. Likewise, there were no significant differences from the controls for target genes *EPSPS*, *EXT2* and *P4HA1*, and only weak (*P* > 0.01) effects for *CAD*, *GAUT12*, *HB3*, *IQD10*, *RWA2*, *SHMT* and *VND6* (Fig. 3). None of these lines were significantly different from controls based on the Tukey’s HSD tests (Fig. 5).

The *DUF231* TOP line flushed significantly earlier and had also increased height growth compared to controls (Fig. 5a, b). This gene belongs to the Trichome Birefringence-Like (TBL) gene family [49]. Members of the TBL family are responsible for *O*-acetylation of hemicelluloses in *Arabidopsis thaliana*, and knockouts of these genes show altered cell wall phenotypes, including reduced cellulose crystallinity and decreased esterification [50]. Although the mechanisms of early bud flush remain to be determined, one might speculate that increased cell wall permeability in the *DUF231* overexpression line facilitates diffusion of growth-promoting signals such as the *FT1* protein into the dormant bud to promote resumption of growth, a scenario that is consistent with the central role of glucan hydrolases in releasing dormancy in *Populus* [51, 52]. Other target genes also showed a trend toward early bud flush, including *DUF266*, *EXT1*, *PFD2*, and *VND6* (Figs. 3, 5b). Each of these could also have impacts on cell wall permeability, so a similar explanation for this trend could apply in each of these cases.

One of the comparator lines of the *DUF266* target gene also showed significantly decreased internode length in addition to early bud flush (Fig. 5b, c). Interestingly, total height was barely reduced and volume index was slightly higher than the controls, due to an increase in stem diameter. It is worth noting that the bud flush phenotype could not have had a direct effect on the yield and growth values for this specific study, since it was measured in 2017 and the growth reported here occurred prior to this. Therefore, early bud flush could not have compensated for the observed reduction in internode length. This gene is a putative glycosyltransferase with direct impacts on cellulose biosynthesis. The proportion of cellulose and cellulose polymerization were both substantially elevated in stems of these transgenic lines in greenhouse studies [53]. However, the molecular mechanisms underlying these phenotypic effects have yet to be determined, so the reduced internode length and enhanced stem diameter remain to be explained.

Overexpression lines of *PFD2* showed marginal significance for five traits, related to biomass and bud flush, pointing at a possible subtle trend (Fig. 3). Indeed, the two lines showed a significantly reduced volume index compared to the controls (Fig. 5d). The closest ortholog of this gene in *Arabidopsis thaliana* is *AT3G22480* [54], which is part of the heterohexameric prefoldin complex, comprised of PFD1-6. Other members of this complex, specifically *PFD3* and *PFD5*, bind to the *DELLA* protein, which mediates their levels in the cytosol, where the prefoldin complex is responsible for proper cortical microtubule formation [55]. *DELLA* proteins are diurnally regulated by gibberellin (GA) phytohormones, and their interactions with the prefoldin complex provides a possible mechanism for regulating cell wall expansion and anisotropic growth based on the formation and orientation of cortical microtubules [55, 56]. Overexpression of one member of the prefoldin complex may have disrupted this regulation, leading to reduced volume growth in the field. It is unclear why the opposite effect was seen in greenhouse studies (Table 1; unpublished observations), but since *DELLA* proteins are responsible for mediating photomorphogenesis, light quality (e.g., the red:far-red ratio), could be a factor [15, 57].

## Conclusions

Overall the results of this trial reflect well upon the transgenic lines that have emerged from the intensive screening process conducted by the BESC. More than 500 gene targets have been evaluated in numerous greenhouse and growth chamber trials to identify genes with positive effects on sugar release in a high-throughput assay using thermochemical pretreatment and enzymatic hydrolysis [14]. Most lines have not shown any significant reductions in growth or tolerance of biotic or abiotic stresses in this field trial, despite several substantial challenges, including large outbreaks of the cottonwood twig borer and *Melampsora* leaf rust, as well as a late frost event. This is in contrast to some previous field studies of *Populus* trees with modified cell wall characteristics that show reduced yield in the field, including down-regulation of *4CL* [23, 24], and down-regulation of cinnamoyl-CoA reductase [36]. One note of caution is that these trees have not yet experienced substantial drought stress due to irrigation in the first year, and mechanical stresses were mitigated by the use of tree collars and stakes during the establishment period. Irrigation and fertilization has been discontinued and the stakes have been removed, so it will be interesting to see if there are differential responses to drought, insects, and pathogens under more stressful conditions. It will also be important to determine if cell wall characteristics and enhanced saccharification efficiency persist in the field. Finally, a replicate trial is underway in Georgia, so there will be an opportunity to evaluate genotype-by-environment interactions for these lines, which have proven to be important for other cell wall modifications, such as *4CL* down-regulation [24]. Nevertheless, this first year performance is a positive step toward the development of feedstocks that are optimized for consolidated bioprocessing for biofuel production.

## Additional files

**Additional file 1.** Heatmaps for trait predicted values on each of the coordinates of the trial using a thin-plate spline correction model. Note that the color scale ranges from twice the standard deviation over the mean of the trait observed values to twice the standard deviation below the mean, to reflect the proportion of trait variance accounted for by the model. Traits represented are (A) internode length, (B) height growth, (C) quadratic mean diameter, (D) volume index, (E) height to first branch, (F) number of branches, (G) stem sinuosity, (H) stem length-height ratio, (I) apical index, (J) trunk section eccentricity, (K) bud set, (L) bud flush, (M) frost damage, (N) *Melampsora* severity, and (O) overall herbivory.

**Additional file 2.** Bar plots of several measured traits after TPS correction. Bars correspond to a random subsample of empty-vector trees (very light grey), the wild type (light grey), and the 29 transgenic lines grouped by genes. Dark grey indicates Comparator lines and very dark grey indicates TOP lines. Error bars represent 95% confidence intervals. Asterisks indicate Tukey’s HSD mean difference significance (*α* = 0.05) between the marked transgenic line and the empty-vector control. Traits depicted are (A) total height, (B) quadratic mean diameter, (C) height to first branch, (D) number of branches, (E) stem sinuosity, (F) stem length-height ratio, (G) apical index, (H) trunk section eccentricity, (I) bud set, (J) frost damage, (K) *Melampsora* severity, (L) overall herbivory, and (M) twig borer incidence.

## Abbreviations

BESC: BioEnergy Science Center
ANOVA: analysis of variance
SD: standard deviation
TPS: thin-plate spline
Tukey’s HSD: Tukey’s honest significant difference
*4CL*: 4-hydroxycinnamoyl-CoA Ligase
*CAD*: cinnamyl alcohol dehydrogenase
*DUF231*: domain of unknown function 231
*DUF266*: domain of unknown function 266
*EPSPS*: 5-enolpyruvylshikimate-3-phosphate synthase
*EXT1*: extensin 1
*EXT2*: extensin 2
*GAUT12*: galacturonosyltransferase 12
*HB3*: HOMEOBOX 3
*IQD10*: isoleucine/glutamine (IQ) 67 domain 10
*P4HA1*: prolyl 4-hydroxylase alpha subunit
*PFD2*: prefoldin domain protein 2
*RWA2*: reduced wall acetylation 2
*SHMT*: serine hydroxymethyltransferase
*VND6*: vascular-related NAC-domain protein 6
