# Supplementary figures and images for: Agronomic performance of Populus deltoides trees engineered for biofuel production
Source: Biotechnol Biofuels. 2017 Nov 30;10:253. doi: 10.1186/s13068-017-0934-6 (PMC5707814; doi:10.1186/s13068-017-0934-6)

(A)

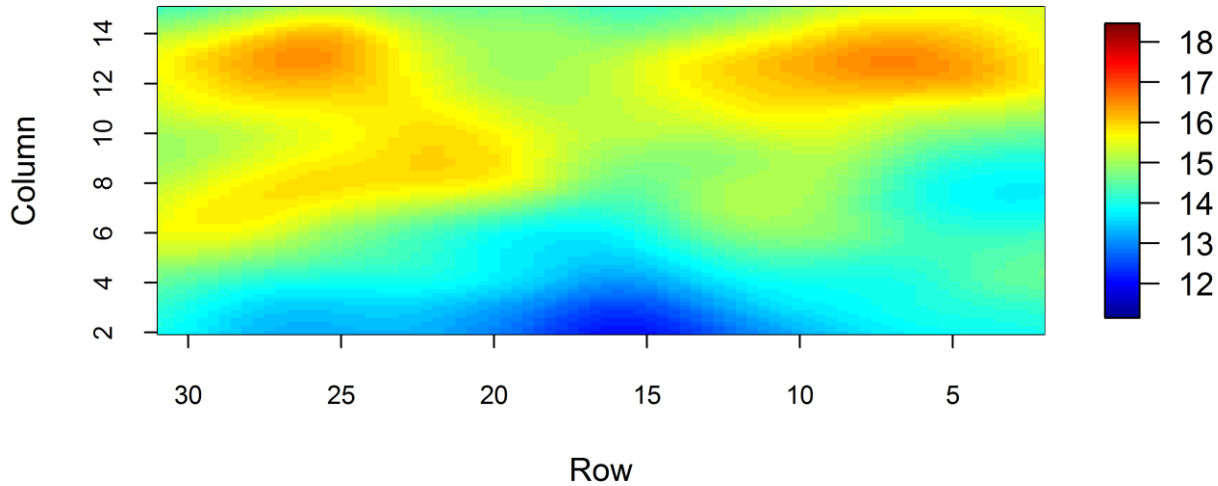

(B)

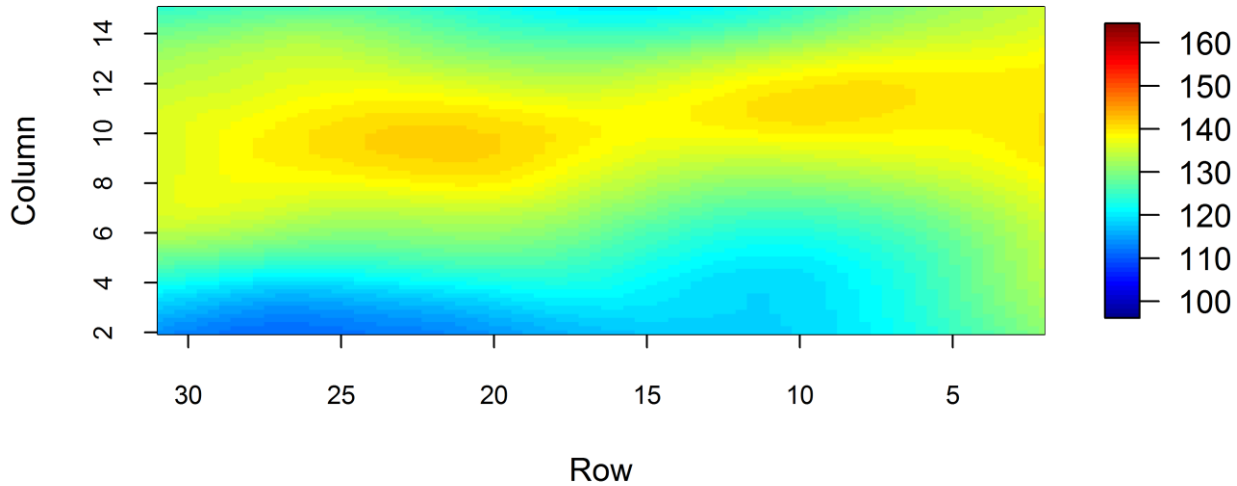

(C)

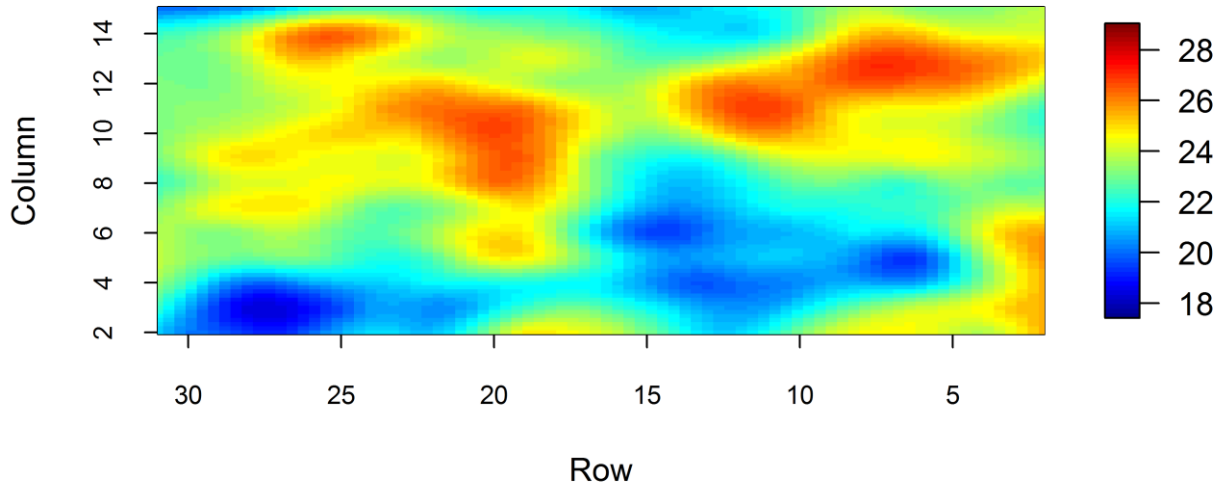

(D)

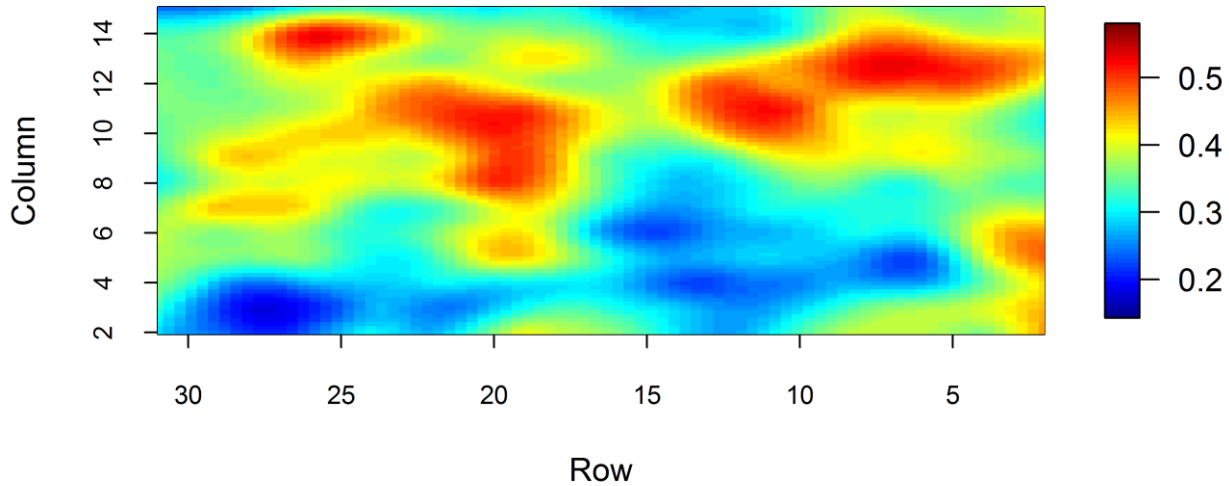

(E)

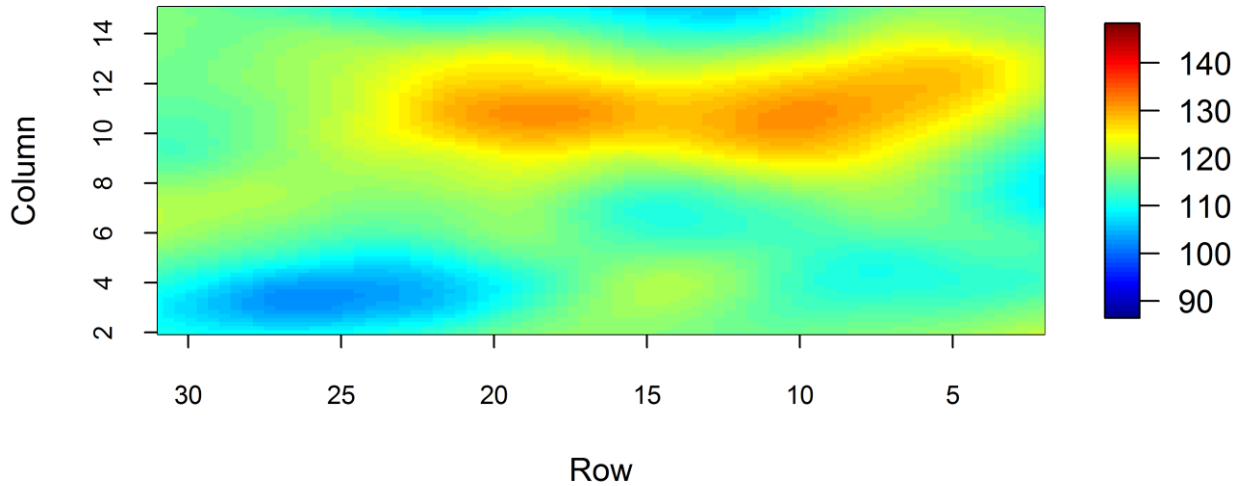

(F)

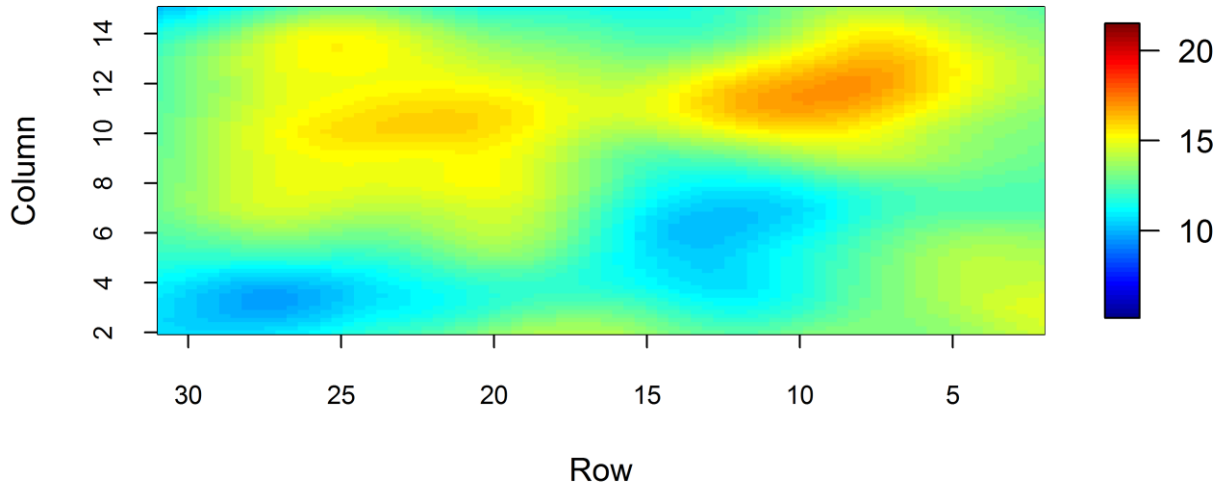

(G)

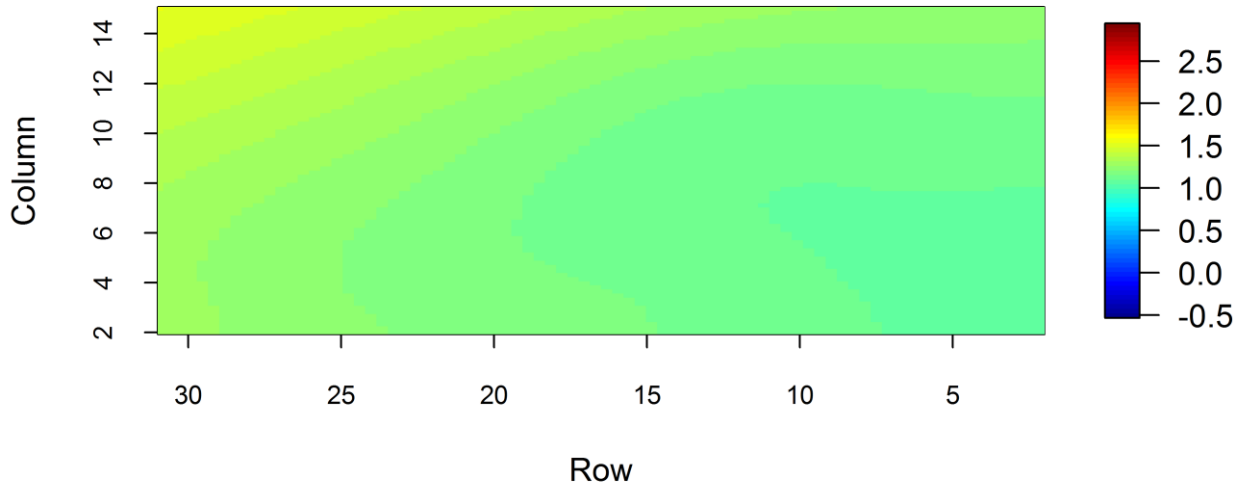

(H)

Column

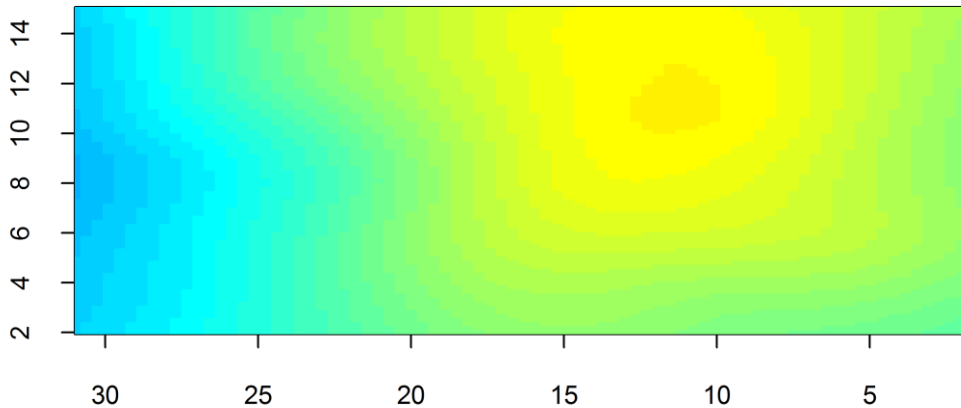

Row

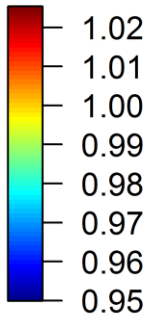

(I)

Column

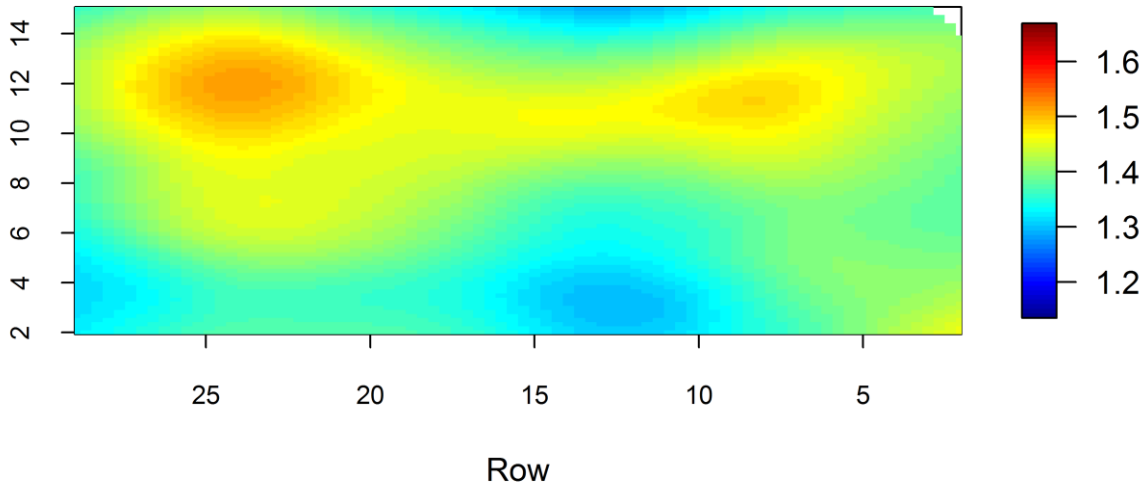

(J)

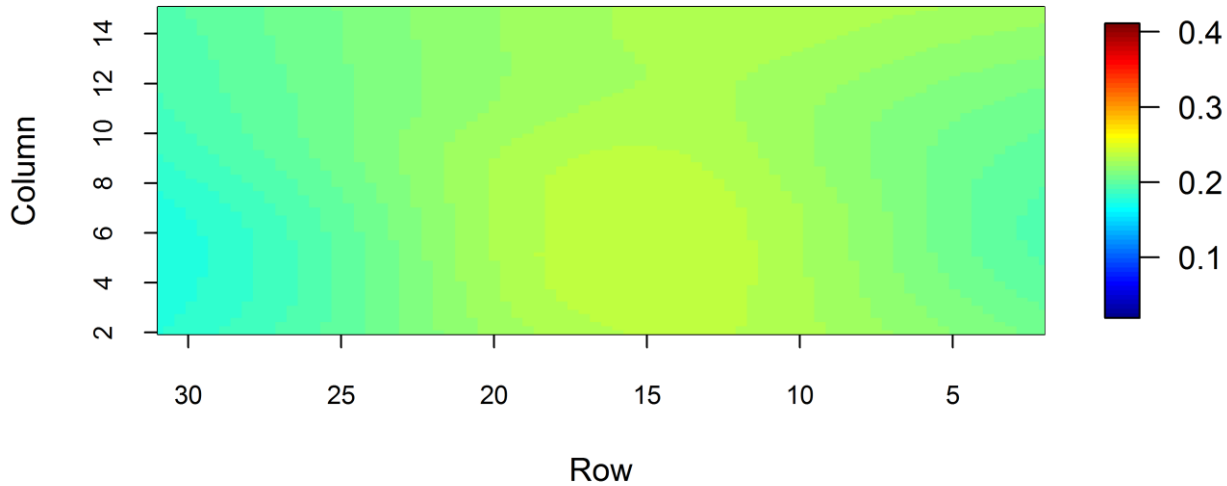

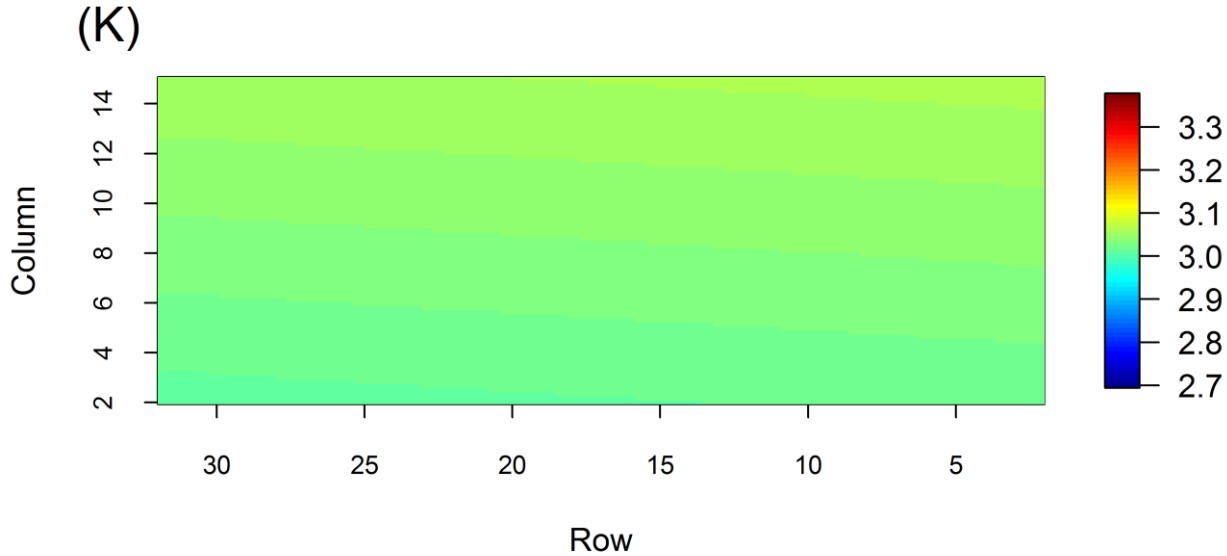

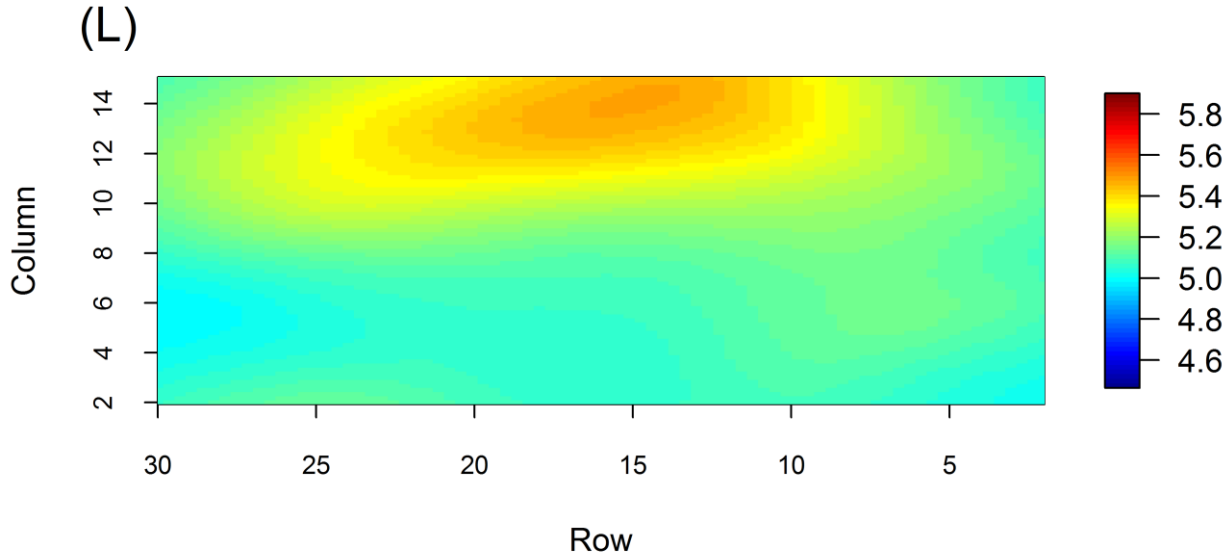

(M)

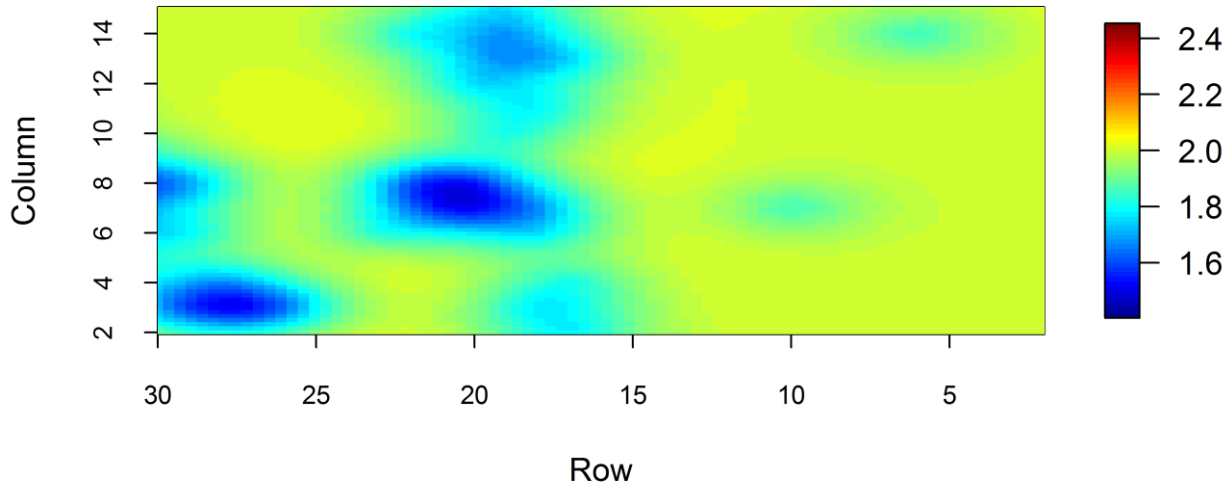

(N)

Column

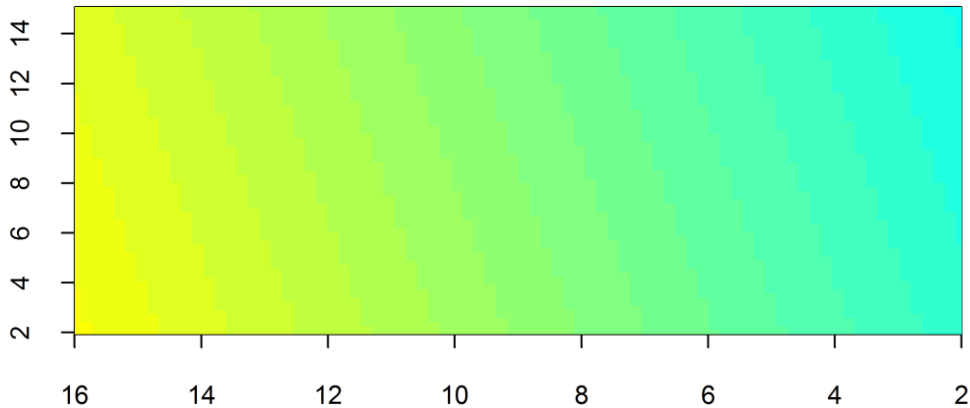

Row

(O)

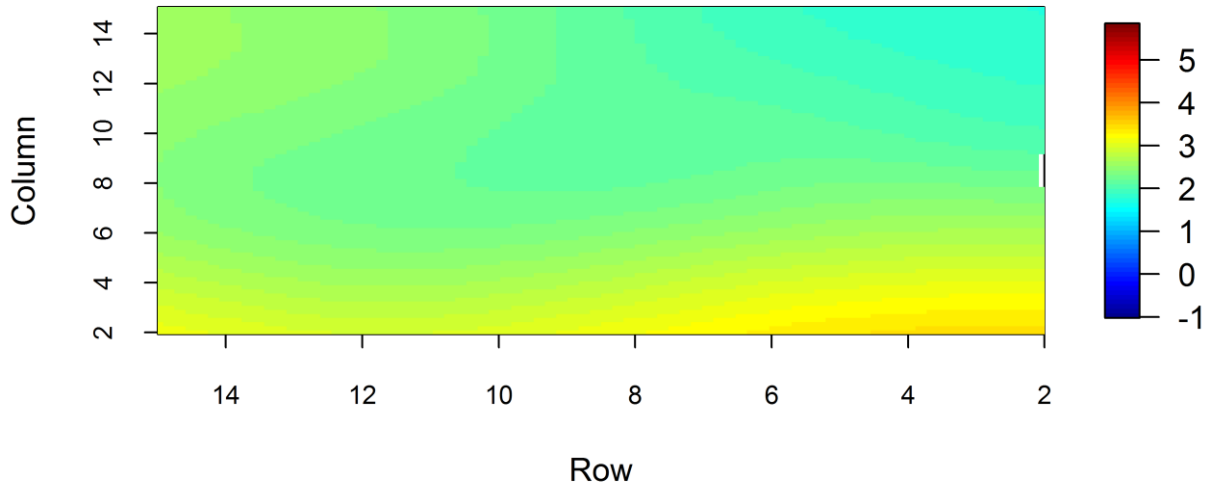

Supplement: Supplementary file 1 — Additional file 1. Heatmaps for trait predicted values on each of the coordinates of the trial using a thin-plate spline correction model. Note that the color scale ranges from twice the standard deviation over the mean of the trait observed values to twice the standard deviation below the mean, to reflect the proportion of trait variance accounted for by the model. Traits represented are (A) internode length, (B) height growth, (C) quadratic mean diameter, (D) volume index, (E) height to first branch, (F) number of branches, (G) stem sinuosity, (H) stem length-height ratio, (I) apical index, (J) trunk section eccentricity, (K) bud set, (L) bud flush, (M) frost damage, (N) Melampsora severity, and (O) overall herbivory. [file 13068_2017_934_MOESM1_ESM.pdf]

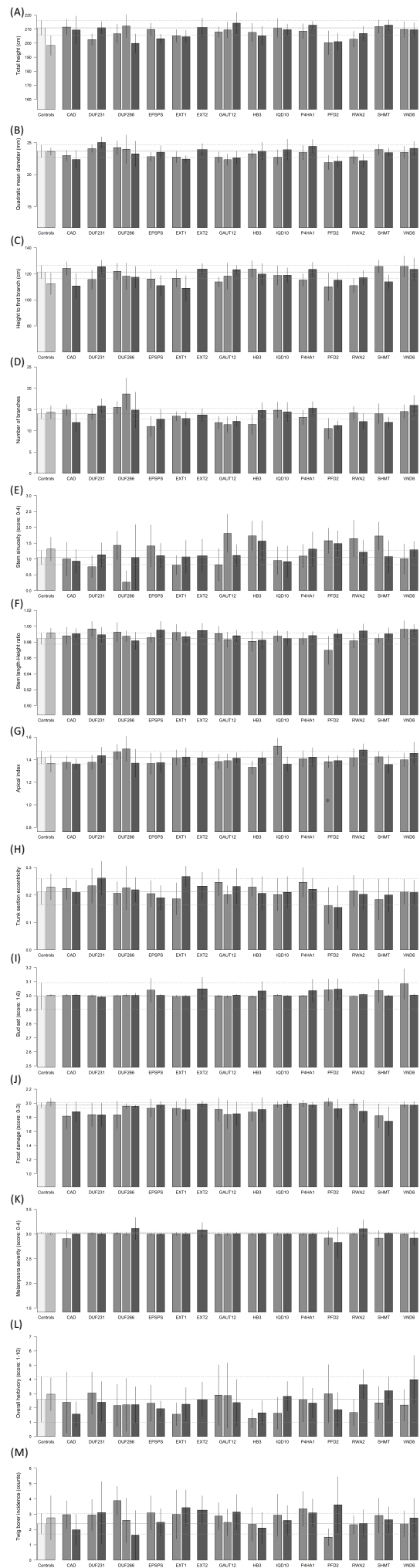

Supplement: Supplementary file 2 — Additional file 2. Bar plots of several measured traits after TPS correction. Bars correspond to a random subsample of empty-vector trees (very light grey), the wild type (light grey), and the 29 transgenic lines grouped by genes. Dark grey indicates Comparator lines and very dark grey indicates TOP lines. Error bars represent 95% confidence intervals. Asterisks indicate Tukey’s HSD mean difference significance (α = 0.05) between the marked transgenic line and the empty-vector control. Traits depicted are (A) total height, (B) quadratic mean diameter, (C) height to first branch, (D) number of branches, (E) stem sinuosity, (F) stem length-height ratio, (G) apical index, (H) trunk section eccentricity, (I) bud set, (J) frost damage, (K) Melampsora severity, (L) overall herbivory, and (M) twig borer incidence. [file 13068_2017_934_MOESM2_ESM.pdf]
